# Drinking alcohol to cope with hyperactive ADHD? Self-reports vs. continuous performance test in patients with ADHD and/or alcohol use disorder

**DOI:** 10.3389/fpsyt.2023.1112843

**Published:** 2023-03-06

**Authors:** Mathias Luderer, Johanna Seidt, Sarah Gerhardt, Sabine Hoffmann, Sabine Vollstädt-Klein, Andreas Reif, Esther Sobanski

**Affiliations:** ^1^Department of Psychiatry, Psychosomatic Medicine and Psychotherapy, University Hospital, Goethe University Frankfurt, Frankfurt, Germany; ^2^Department of Psychiatry and Psychotherapy, Central Institute of Mental Health, Medical Faculty Mannheim, Heidelberg University, Mannheim, Germany; ^3^Department of Addictive Behavior and Addiction Medicine, Central Institute of Mental Health, Medical Faculty Mannheim, Heidelberg University, Mannheim, Germany; ^4^Department of Biostatistics, Central Institute of Mental Health, Medical Faculty Mannheim, Heidelberg University, Mannheim, Germany; ^5^Department of Child and Adolescent Psychiatry and Psychotherapy, Lucerne Cantonal Hospital, Lucerne, Switzerland; ^6^Department of Child and Adolescent Psychiatry and Psychotherapy, University Medical Center Mainz, Mainz, Germany

**Keywords:** attention deficit/hyperactivity disorder (ADHD), alcohol use disorder (AUD), quality of life, continuous performance test, motor activity (MA), mind wandering

## Abstract

**Rationale:**

Attention deficit/hyperactivity disorder (ADHD) is common in alcohol use disorder (AUD). Continuous performance tests (CPTs) allow to measure ADHD related deficits in a laboratory setting. Most studies on this topic focused on CPTs measuring inattention or impulsivity, disregarding hyperactivity as one of the core symptoms of ADHD.

**Methods:**

We examined *N* = 47 in three groups (ADHD *N* = 19; AUD *N* = 16; ADHD + AUD *N* = 12) with questionnaires on ADHD core symptoms, executive functioning (EF), mind wandering, and quality of life (QoL). *N* = 46 (ADHD *N* = 16; AUD *N* = 16; ADHD + AUD *N* = 14) were examined with a CPT (QbTest^®^) that also measures motor activity objectively.

**Results:**

Inattention and impulsivity were significantly increased in AUD vs. ADHD and in AUD vs. ADHD + AUD. Hyperactivity was significantly higher in ADHD + AUD vs. ADHD and ADHD + AUD vs. AUD, but not in ADHD vs. AUD. EF was lower in both ADHD groups vs. AUD. Mind wandering was increased in both ADHD groups vs. AUD. QoL was significantly lower in ADHD + AUD compared to AUD. In contrast, results of the QbTest were not significantly different between groups.

**Conclusion:**

Questionnaires are more useful in assessing ADHD core symptoms than the QbTest^®^. Hyperactivity appears to be a relevant symptom in ADHD + AUD, suggesting a possible pathway from ADHD to AUD. The lower QoL in ADHD + AUD emphasizes the need for routine screening, diagnostic procedures and treatment strategies for this patient group.

## 1. Introduction

Attention deficit/hyperactivity disorder (ADHD) has a prevalence rate of 7.1% in children and adolescents (1) and leads to ongoing symptoms and impairment at least until young adulthood in about 90% of cases (2). Adolescents with ADHD are at increased risk for early initiation and quick escalation of substance use (3–5).

In adults with alcohol use disorder (AUD), comorbid ADHD is common with prevalence rates between 16 and 21% (5–7). However, ADHD is often under-diagnosed in AUD despite its negative effect on adherence and outcome (5).

International expert consensus recommends routine screening for ADHD in patients with substance use disorders (SUDs) (8), although ADHD screening questionnaires show decreased validity in AUD (9). In any case, additional diagnostic assessment is needed to verify ADHD diagnosis, while so far only one diagnostic interview for ADHD has been validated in AUD (5).

The high prevalence of ADHD in AUD (5–7) and low detection rate (5) affect the outcome for the individual patient but also the results of basic human and clinical research. AUD studies most likely included an uncertain number of undetected ADHD cases (10).

Sensitive methods objectively measuring ADHD symptoms could therefore aid in clinical and scientific settings.

Continuous performance tests (CPTs) to measure ADHD symptoms have not been studied extensively in the comorbidity of SUD with ADHD (11), but hyperactivity has been suggested as a relevant parameter for ADHD in SUD (12).

Our study aimed to investigate whether objective measurements or self-rating scales on inattention, impulsivity, and hyperactivity would show differences between patients with ADHD, AUD, and ADHD + AUD.

## 2. Materials and methods

Participants were 18–64 years old and patients in treatment at the Central Institute of Mental Health (Mannheim, Germany).

### 2.1. Inclusion criteria

Alcohol use disorder was diagnosed by trained masters- or medical-degreed personnel according to ICD-10 alcohol dependence (13); participants had to be abstinent for at least 5 days prior to study inclusion (completed detoxification).

Attention deficit/hyperactivity disorder was diagnosed by trained masters- or medical-degreed personnel according to clinical guidelines (14), based on DSM-5 criteria for adult ADHD (15). If available, structured interviews, school records and informants’ ratings were used for the diagnostic assessment.

### 2.2. Exclusion criteria

For the groups AUD or ADHD, individuals with a diagnosis of a mental disorder within the last year or intake of psychotropic medication within the last 3 days were excluded. Stable medication with fluoxetine was considered acceptable in one participant in the ADHD group (self-reports only). Participants in the ADHD group were allowed to take prescribed stimulants, but not the day when the QbTest was conducted.

Psychiatric comorbidity did not lead to an exclusion for the ADHD + AUD group, as psychiatric comorbidity in individuals with ADHD and SUD is remarkably high (10, 16–18).

#### 2.2.1. AUD group

Participants in the AUD group had to screen negative for ADHD in three different questionnaires: Wender Utah Rating Scale (19), ADHD self-report scale (20), Adult ADHD Self-Report Scale (21).

#### 2.2.2. ADHD group

Participants in the ADHD group had to screen negative (cut-off <8) for AUD in the Alcohol Use Disorder Identification Test (22).

#### 2.2.3. Exclusion criteria for all groups

• Cocaine/amphetamine/opioid dependence lifetime
• Lifetime diagnosis of delusional disorders, schizophrenia, or bipolar disorder
• Severe physical illness

The Ethics Committee of the Medical Faculty Mannheim, Heidelberg University, Germany approved the study beforehand (approval number 2013-530 N-MA). All participants provided written informed consent and the study was in accordance with the Declaration of Helsinki.

### 2.3. QbTest^®^

This commercially available CPT comprises of a 1-back task while measuring head movements with an infrared camera and a reflector attached to a headband. The QbTest^®^ provides raw scores for motor activity, inattention and impulsivity. From these raw scores, the cardinal parameters Qb-activity, Qb-inattention, and Qb-impulsivity are derived by performing a principal component analysis. These parameters are transformed into normally distributed Q-scores implicating information about the difference between the individual raw score compared to scores of a gender- and age-controlled group (23).

### 2.4. Self-report scales

#### 2.4.1. ADHD Self-Rating Scale

ADHD Self-Rating Scale (ADHD-SR) consists of 18 items (DSM criteria for ADHD). A total score as well as sub-scores on hyperactivity, impulsivity, and inattention can be calculated (20, 24).

#### 2.4.2. Barratt Impulsivity Scale

The 30 items of the Barratt Impulsivity Scale (BIS-11) assess trait impulsivity [attentional (e.g., distraction), motor, and non-planning] (25).

#### 2.4.3. Quality of Life Enjoyment and Satisfaction Questionnaire

The 16 items of the Quality of Life Enjoyment and Satisfaction Questionnaire (Q-LES-Q) measure quality of life (QoL) as the degree of enjoyment and satisfaction experienced in various areas of daily functioning (26).

#### 2.4.4. Behavior Rating Inventory of Executive Function – Adult Version

Executive functioning (EF) summarizes a set of advanced cognitive abilities that allow cognitive, emotional, and motor control to create goals, create plans to achieve the goals and stick to the plan until the goals are achieved. Higher scores in the 75 items Behavior Rating Inventory of Executive Function-Adult Version (BRIEF-A) mean more EF deficits (27).

#### 2.4.5. Mind Excessively Wandering Scale

Unintentional mind wandering is common in ADHD and higher frequency of mind wandering is associated with increased functional impairment and related to lower EF (28). The Mind Excessively Wandering Scale (MEWS) consists of 15 items (29, 30).

### 2.5. Statistical analysis

Data were analyzed with IBM SPSS Statistics for Windows, Version 27.0. The distribution of gender was compared using Fisher’s exact test. An analysis of variance (ANOVA) was calculated to compare the mean age between groups. Since cardinal Qb-parameters were already controlled for sex and age, we applied an ANOVA, using Bonferroni correction for multiple testing. A one-way analysis of covariance (ANCOVA) was conducted on the questionnaire scores controlling for age and gender, using a Bonferroni correction for multiple testing. A *p*-value < 0.05 was used as significance level for all calculations.

## 3. Results

Complete data on the self-report scales were available for *N* = 47 participants (ADHD *N* = 19; AUD *N* = 16; ADHD + AUD *N* = 12) and were included in this analysis. For the QbTest^®^, we included *N* = 46 (ADHD *N* = 16; AUD *N* = 16; ADHD + AUD *N* = 14).

Age and gender were significantly different between groups, see Table 1.

**TABLE 1:** Basic characteristics.

		AUD	ADHD	ADHD + AUD	*p*-Value		
					**AUD vs.** **ADHD**	**AUD vs.** **ADHD + AUD**	**ADHD vs.** **ADHD + AUD**
QbTest	% male	94%	75%	50%	0.333^a^	0.012^a^	0.257^a^
	Age	44.4 (12.1)	29.4 (7.8)	40.0 (11.7)	<0.001^b^	0.269^b^	0.009^b^
Self-report scales	% male	94%	68%	50%	0.092^a^	0.011^a^	0.452^a^
	Age	44.0 (12.5)	30.4 (9.7)	39.7 (11.7)	0.001^b^	0.314^b^	0.030^b^

^a^Fisher’s exact test.

^b^ANOVA with *a priori* contrasts between groups.

### 3.1. QbTest^®^

**FIGURE 1:**
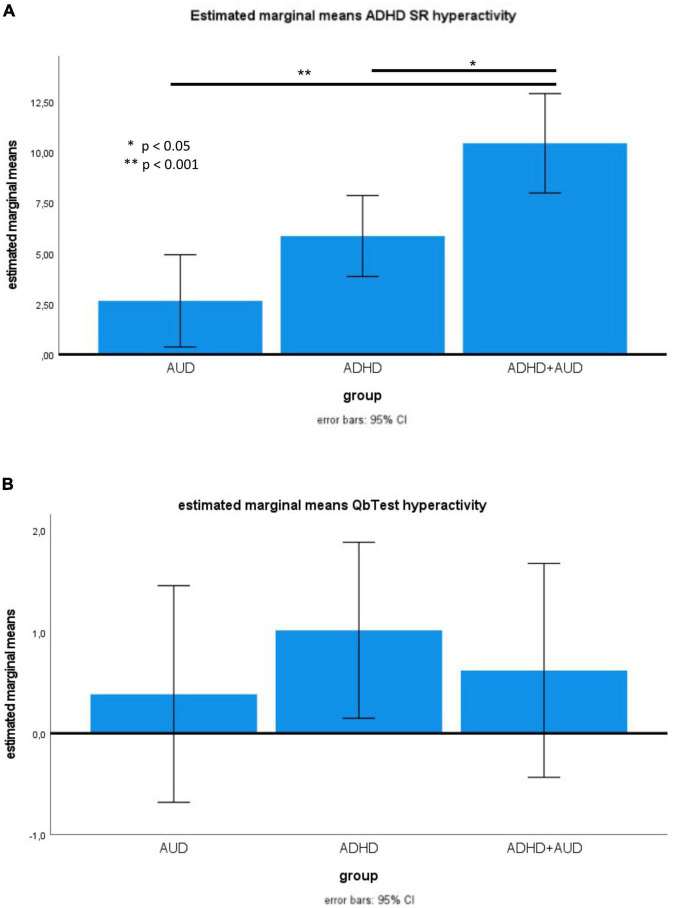
**(A)** Estimated marginal means of ADHD-SR hyperactivity. **(B)** Means of Qb activity.

One-way ANOVA with Bonferroni *post-hoc* correction showed no statistically significant differences between groups for all QbTest results (see Table 2 and Figure 1B).

**TABLE 2:** QbTest.

	AUD	ADHD	ADHD + AUD	*F*	df	*p*-Value			
	***N* = 16**	***N* = 16**	***N* = 14**			**Overall** **model**	**AUD vs.** **ADHD**	**AUD vs.** **ADHD + AUD**	**ADHD vs.** **ADHD + AUD**
Hyperactivity	−0.69 (1.57)	1.09 (1.32)	0.44 (1.77)	2.224	2	0.120	0.124	1.0	0.775
Impulsivity	0.19 (1.52)	0.79 (1.09)	0.58 (1.18)	0.897	2	0.415	0.581	1.0	1.0
Inattention	0.21 (1.01)	0.83 (0.75)	0.34 (1.5)	1.359	2	0.268	0.370	0.716	1.0

Means of QbTest cardinal parameters (ANOVA, Bonferroni *post-hoc* correction); standard deviation in brackets.

### 3.2. Questionnaires

One-way analysis of covariance with Bonferroni *post-hoc* correction showed statistically significant differences between groups: inattention, impulsivity (both measured with BIS-11 and ADHD-SR), mind wandering, and EF were significantly different in ADHD vs. AUD and in ADHD + AUD vs. AUD, but not in ADHD + AUD vs. ADHD.

In contrast, hyperactivity was significantly increased in ADHD + AUD vs. ADHD, and in ADHD + AUD vs. AUD (Figure 1A), while the numerical difference in ADHD vs. AUD did not reach significance. QoL was significantly lower in ADHD + AUD vs. AUD, but no further statistically significant group differences emerged (Table 3).

**TABLE 3:** Self-report scales.

	AUD	ADHD	ADHD + AUD	*F*	df	Partial η^2^	*p*-Value			
	***N* = 17**	***N* = 19**	***N* = 12**				**Overall** **model**	**AUD vs.** **ADHD**	**AUD vs.** **ADHD + AUD**	**ADHD vs.** **ADHD + AUD**
ADHD-SR hyperactivity	2.6 (1.1)	5.8 (1.0)	10.5 (1.1)	11.997	2	0.358	<0.001	0.111	<0.001	0.015
ADHD-SR inattention	3.1 (1.1)	17.9 (1.0)	16.3 (1.3)	47.082	2	0.687	<0.001	<0.001	<0.001	1.0
ADHD-SR impulsivity	1.3 (0.8)	5.8 (0.8)	6.2 (0.9)	9.582	2	0.308	<0.001	0.002	0.001	1.0
BIS-11 impulsivity	63.5 (2.8)	75.8 (2.6)	76.1 (3.1)	5.801	2	0.216	0.006	0.013	0.017	1.0
MEWS mind wandering	10.9 (2.1)	26.6 (1.9)	27.4 (2.3)	18.177	2	0.458	<0.001	<0.001	<0.001	1.0
BRIEF executive functioning	103.2 (5.0)	148.3 (4.6)	148.9 (5.6)	24.589	2	0.534	<0.001	<0.001	<0.001	1.0
Q-LES-Q quality of life	50.4 (1.8)	45.5 (1.6)	39.4 (2.0)	8.198	2	0.276	<0.001	0.196	0.001	0.071

Estimated marginal means of self-report scales adjusted for gender and age (ANCOVA, Bonferroni *post-hoc* correction); standard deviation in brackets.

## 4. Discussion

To our knowledge, this is the first study directly comparing individuals with AUD and/or ADHD objectively measuring hyperactivity and other core ADHD symptoms (inattention, impulsivity).

ADHD + AUD showed increased hyperactivity in self-reports. Other ADHD related deficits also showed differences for ADHD groups (ADHD + AUD or ADHD) vs. AUD. However, we found no group differences for the QbTest parameters.

Substance use disorder and ADHD show comparable inattentive and impulsive behavior in CPTs, but hyperactivity was identified as a promising parameter to distinguish ADHD from SUD (11). One study used certain measures of motor impulsivity (e.g., pressing buttons too quickly or randomly) as a proxy for hyperactivity, and found a significant difference between ADHD (with or without SUD) and SUD, although the effect size was small (31).

Attention deficit/hyperactivity disorder and healthy controls significantly differ in all ADHD core symptoms as measured by the QbTest^®^ (32). Although sensitivity and specificity for the QbTest^®^ were decreased in clinical samples, most studies still found differences on a group level (33–36), especially regarding hyperactivity (36). Thus, ADHD remains a clinical diagnosis (14).

This contrasts with the results of our study. Here, the QbTest^®^ did not show any differences between groups regarding objectively measured ADHD core symptoms.

In ADHD, new and interesting situations with low distraction – as in (laboratory) research – often lead to a temporary decrease in symptoms including hyperactivity (37). Functional imaging studies on AUD and ADHD showed similar impulsivity and resting state brain networks (38), but brain activation during inhibition tasks depended on ADHD and AUD severity (39). These findings, together with our results, suggest that in ADHD a less effective brain network compensates impulsivity, which might be more vulnerable to distractors or a general increased level of stress in real life (40), but not in a controlled laboratory setting. This might also apply to other ADHD symptoms.

Larger samples might be needed to detect group differences in ADHD related deficits (11, 31) due to a large overlap of affected domains in ADHD and AUD (41). However, larger samples often lead to more heterogeneous groups (31). This might affect study results, as for example heavy cannabis use has been associated with more ADHD related deficits during a CPT (42). In our study, we investigated a more homogenous sample (e.g., exclusion of other SUDs).

Of note, self-report questionnaires already showed significant and group specific differences at the given sample size.

Hyperactivity decreases during adolescence (43). In adults, a feeling of inner restlessness can be the main hyperactive symptom (44) and thus can only be assessed by self-report. However, persisting hyperactivity has been associated with severe comorbidity such as SUD (45). This is confirmed by our study, as ADHD + AUD reported higher hyperactivity than AUD or ADHD alone. Adolescents with ADHD and comparably high hyperactivity might be more prone to drink alcohol to cope with their hyperactivity and develop an AUD subsequently. ADHD did not differ from AUD regarding self-reported hyperactivity, implicating that increased hyperactivity is a possible pathway from childhood ADHD to development of AUD later in adolescence or early adulthood, probably to reduce unpleasant hyperactivity or restlessness (46). As we did not assess comorbidity, hyperactivity could also be driven by co-occurring mental health problems such as emotional dysregulation (10), traumatic experiences (17, 47) or trauma-related disorders (16, 17).

Our results show that individuals with ADHD (with or without AUD) are more impulsive than individuals with AUD only. Decades of research have linked AUD to increased impulsivity (48). Impulsivity is either increased in AUD (but even more in ADHD + AUD) or only increased in AUD + ADHD. Supporting the latter, impulsivity is mainly increased in individuals with “early onset” AUD (49). Since the age of onset of AUD is almost 10 years earlier in patients with ADHD (5), many ADHD cases might be hidden in the term “early onset” AUD.

Although impulsivity and alcohol consumption in adolescence negatively affect each other leading to early AUD (10, 50, 51), we did not find differences in impulsivity between ADHD and ADHD + AUD. This is probably due to a ceiling effect, as the impulsivity scores in both ADHD groups corresponded to the scores of in-patients in a forensic setting (52).

Attention deficit/hyperactivity disorder or ADHD + AUD had more deficits in EF compared to AUD, which is in line with recent findings (53). EF deficits are associated with an increased risk for treatment drop-out in AUD (54) and SUD + ADHD (55) and lower QoL in ADHD (56).

Mind wandering (MW) was increased in ADHD (with or without AUD) compared to AUD. MW is associated with lower EF, lower QoL and more severe ADHD (28, 57), and an increased risk for traffic accidents (58, 59). Alcohol consumption increases MW but decreases individual awareness for MW (28). Since individuals with ADHD are at increased risk for traffic accidents, especially with co-occurring substance use (40), reducing MW might be relevant especially in ADHD + AUD.

Attention deficit/hyperactivity disorder or AUD is associated with low QoL (60, 61) but successful treatment improves QoL (62, 63). In our study, ADHD + AUD had lower QoL vs. AUD, while ADHD did not differ from AUD. We conclude that co-occurrence of AUD and ADHD has a significant impact on QoL in AUD. QoL is an important outcome parameter in AUD (64) and studies on interventions in ADHD + AUD might also address improvement of QoL.

## 5. Conclusion

In conclusion, self-report scales on core symptoms of ADHD as well as on ADHD related deficits showed significant and specific group differences between ADHD, AUD, and ADHD + AUD, while a CPT did not show any differences between groups.

Hyperactivity was confirmed to be a relevant symptom in ADHD + AUD, suggesting a possible pathway from ADHD to AUD. The lower QoL in ADHD + AUD compared to AUD emphasizes the need for routine ADHD screening, diagnostic procedures and treatment strategies in patients with AUD.

## Data availability statement

The datasets presented in this article are not readily available because the data contain information that would compromise research participant consent. We will provide data upon direct request by research colleagues following current data protection guidelines. Requests to access the datasets should be directed to ML, mathias.luderer@kgu.de.

## Ethics statement

The studies involving human participants were reviewed and approved by the Ethics Committee of the Medical Faculty Mannheim, Heidelberg University, Heidelberg, Germany. The patients/participants provided their written informed consent to participate in this study.

## Author contributions

ML and ES designed the study. ML, JS, and SG collected the sample. ML performed the literature review, conducted the analyses together with JS, and wrote the initial version of this manuscript. All authors contributed to the article and approved the submitted version.

## References

[1] Thomas R Sanders S Doust J Beller E Glasziou P. Prevalence of attention-deficit/hyperactivity disorder: a systematic review and meta-analysis. *Pediatrics.* (2015) 135:e994–1001. 10.1542/peds.2014-3482 25733754

[2] Sibley M Arnold L Swanson J Hechtman L Kennedy T Owens E Variable patterns of remission from ADHD in the multimodal treatment study of ADHD. *Am J Psychiatry.* (2022) 179:142–51. 10.1176/appi.ajp.2021.21010032 34384227 PMC8810708

[3] Molina B Howard A Swanson J Stehli A Mitchell J Kennedy T Substance use through adolescence into early adulthood after childhood-diagnosed ADHD: findings from the MTA longitudinal study. *J Child Psychol Psychiatry.* (2018) 59:692–702. 10.1111/jcpp.12855 29315559 PMC5985671

[4] Vogel T Dom G van de Glind G Studer J Gmel G Strik W Is attention deficit/hyperactivity disorder among men associated with initiation or escalation of substance use at 15-month follow-up? A longitudinal study involving young Swiss men. *Addiction.* (2016) 111:1867–78. 10.1111/add.13422 27061514 PMC5215781

[5] Luderer M Sick C Kaplan-Wickel N Reinhard I Richter A Kiefer F Prevalence estimates of ADHD in a sample of inpatients with alcohol dependence. *J Atten Disord.* (2020) 24:2072–83. 10.1177/1087054717750272 29308693

[6] Daigre C Roncero C Rodriguez-Cintas L Ortega L Lligona A Fuentes S Adult ADHD screening in alcohol-dependent patients using the wender-utah rating scale and the adult ADHD self-report scale. *J Atten Disord.* (2015) 19:328–34. 10.1177/1087054714529819 24743975

[7] Roncero C Ortega L Perez-Pazos J Lligona A Abad A Gual A Psychiatric comorbidity in treatment-seeking alcohol dependence patients with and without ADHD. *J Atten Disord.* (2015) 23:1497–504. 10.1177/1087054715598841 26269096

[8] Crunelle C van den Brink W Moggi F Konstenius M Franck J Levin F International consensus statement on screening, diagnosis and treatment of substance use disorder patients with comorbid attention deficit/hyperactivity disorder. *Eur Addict Res.* (2018) 24:43–51. 10.1159/000487767 29510390 PMC5986068

[9] Luderer M Kaplan-Wickel N Richter A Reinhard I Kiefer F Weber T. Screening for adult attention-deficit/hyperactivity disorder in alcohol dependent patients: underreporting of ADHD symptoms in self-report scales. *Drug Alcohol Depend.* (2019) 195:52–8. 10.1016/j.drugalcdep.2018.11.020 30583265

[10] Luderer M Ramos Quiroga J Faraone S Zhang James Y Reif A. Alcohol use disorders and ADHD. *Neurosci Biobehav Rev.* (2021) 128:648–60. 10.1016/j.neubiorev.2021.07.010 34265320

[11] Slobodin O. The utility of the CPT in the diagnosis of ADHD in individuals with substance abuse: a systematic review. *Eur Addict Res.* (2020) 26:283–94. 10.1159/000508041 32535592

[12] Baggio S Hasler R Giacomini V El-Masri H Weibel S Perroud N Does the continuous performance test predict ADHD symptoms severity and ADHD presentation in adults? *J Atten Disord.* (2020) 24:840–8. 10.1177/1087054718822060 30654686

[13] World Health Organization (WHO). *The ICD-10 classification of mental and behavioural disorders : clinical descriptions and diagnostic guidelines.* Geneva: World Health Organization (1992).

[14] AWMF. *Long version of the interdisciplinary evidence- and consensus-based (S3) guideline “attention-deficit/hyperactivity disorder (ADHD) in children, adolescents and adults”.* (2020). Available online at: https://register.awmf.org/assets/guidelines/028_D_G_f_Kinder-_und_Jugendpsychiatrie_und_-psychotherapie/028-045eng_S3_ADHS_2020-12.pdf (accessed February 20, 2023).

[15] American Psychiatric Association. *DSM Task force. Diagnostic and statistical manual of mental disorders: DSM-5.* Nevada, VA: American Psychiatric Association (2013). 10.1176/appi.books.9780890425596

[16] Brynte C Aeschlimann M Barta C Begeman A Bäcker A Crunelle C The clinical course of comorbid substance use disorder and attention deficit/hyperactivity disorder: protocol and clinical characteristics of the INCAS study. *BMC Psychiatry.* (2022) 22:625. 10.1186/s12888-022-04259-6 36151539 PMC9502646

[17] Luderer M Reinhard I Richter A Kiefer F Weber T. ADHD is associated with a higher risk for traumatic events, self-reported PTSD, and a higher severity of PTSD symptoms in alcohol-dependent patients. *Eur Addict Res.* (2020) 26:245–53. 10.1159/000508918 32653887

[18] van Emmerik-van Oortmerssen K van de Glind G Koeter M Allsop S Auriacombe M Barta C Psychiatric comorbidity in treatment-seeking substance use disorder patients with and without attention deficit hyperactivity disorder: results of the IASP study. *Addiction.* (2014) 109:262–72. 10.1111/add.12370 24118292 PMC4112562

[19] Retz-Junginger P Retz W Blocher D Weijers H Trott G Wender P [Wender utah rating scale. The short-version for the assessment of the attention-deficit hyperactivity disorder in adults]. *Nervenarzt.* (2002) 73:830–8. 10.1007/s00115-001-1215-x 12215873

[20] Rösler M Retz W Retz-Junginger P Thome J Supprian T Nissen T [Tools for the diagnosis of attention-deficit/hyperactivity disorder in adults. Self-rating behaviour questionnaire and diagnostic checklist]. *Nervenarzt.* (2004) 75:888–95. 10.1007/s00115-003-1622-2 15378249

[21] Kessler R Adler L Gruber M Sarawate C Spencer T Van Brunt D. Validity of the world health organization adult ADHD self-report scale (ASRS) screener in a representative sample of health plan members. *Int J Methods Psychiatric Res.* (2007) 16:52–65. 10.1002/mpr.208 17623385 PMC2044504

[22] Reinert D Allen J. The alcohol use disorders identification test (AUDIT): a review of recent research. *Alcohol Clin Exp Res.* (2002) 26:272–9. 10.1111/j.1530-0277.2002.tb02534.x 11964568

[23] QbTech. *A QbTest plus technical manual.* Gothenburg: QbTech (2010).

[24] Luderer M Kaplan-Wickel N Sick C Richter A Reinhard I Kiefer F ADHS-screening bei alkoholabhängigen. *Der Nervenarzt.* (2019) 90:1156–61. 10.1007/s00115-019-0706-6 30976828

[25] Patton J Stanford M Barratt E. Factor structure of the barratt impulsiveness scale. *J Clin Psychol.* (1995) 51:768–74. 10.1002/1097-4679(199511)51:6<768::AID-JCLP2270510607>3.0.CO;2-1 8778124

[26] Endicott J Nee J Harrison W Blumenthal R. Quality of life enjoyment and satisfaction questionnaire: a new measure. *Psychopharmacol Bull.* (1993) 29:321–6. 10.1037/t49981-000 8290681

[27] Roth R Gioia G. *Behavior rating inventory of executive function–adult version.* Florida: Psychological Assessment Resources Lutz (2005). 10.1037/t86244-000

[28] Biederman J Lanier J DiSalvo M Noyes E Fried R Woodworth K Clinical correlates of mind wandering in adults with ADHD. *J Psychiatr Res.* (2019) 117:15–23. 10.1016/j.jpsychires.2019.06.012 31272014

[29] Mowlem F Skirrow C Reid P Maltezos S Nijjar S Merwood A Validation of the mind excessively wandering scale and the relationship of mind wandering to impairment in adult ADHD. *J Atten Disord.* (2019) 23:624–34. 10.1177/1087054716651927 27255536 PMC6429624

[30] Nakovics H Benoit D Asherson P Luderer M Alm B Vollstädt-Klein S Validation of the german version of the mind excessively wandering scale (MEWS-G). *Fortschr Neurol Psychiatr.* (2021) 89:607–16. 10.1055/a-1362-9743 33657626

[31] Slobodin O Blankers M Kapitány-Fövény M Kaye S Berger I Johnson B Differential diagnosis in patients with substance use disorder and/or attention-deficit/hyperactivity disorder using continuous performance test. *Eur Addict Res.* (2020) 26:151–62. 32074617 10.1159/000506334

[32] Lis S Baer N Stein-en-Nosse C Gallhofer B Sammer G Kirsch P. Objective measurement of motor activity during cognitive performance in adults with attention-deficit/hyperactivity disorder. *Acta Psychiatr Scand.* (2010) 122:285–94. 20199487 10.1111/j.1600-0447.2010.01549.x

[33] Edebol H Helldin L Norlander T. Objective measures of behavior manifestations in adult ADHD and differentiation from participants with bipolar II disorder, borderline personality disorder, participants with disconfirmed ADHD as well as normative participants. *Clin Pract Epidemiol Ment Health.* (2012) 8:134–43. 10.2174/1745017901208010134 23166565 PMC3497060

[34] Hult N Kadesjö J Kadesjö B Gillberg C Billstedt E. ADHD and the QbTest: diagnostic validity of QbTest. *J Atten Disord.* (2018) 22:1074–80. 26224575 10.1177/1087054715595697

[35] Walker A Shores E Trollor J Lee T Sachdev P. Neuropsychological functioning of adults with attention deficit hyperactivity disorder. *J Clin Exp Neuropsychol.* (2000) 22:115–24. 10649550 10.1076/1380-3395(200002)22:1;1-8;FT115

[36] Brunkhorst-Kanaan N Verdenhalven M Kittel-Schneider S Vainieri I Reif A Grimm O. The quantified behavioral test-a confirmatory test in the diagnostic process of adult ADHD? *Front Psychiatry.* (2020) 11:216. 10.3389/fpsyt.2020.00216 32265761 PMC7100366

[37] Kofler M Raiker J Sarver D Wells E Soto E. Is hyperactivity ubiquitous in ADHD or dependent on environmental demands? Evidence from meta-analysis. *Clin Psychol Rev.* (2016) 46:12–24. 10.1016/j.cpr.2016.04.004 27131918 PMC4902796

[38] Farré-Colomés À Gerhardt S Luderer M Sobanski E Kiefer F Vollstädt-Klein S. Common and distinct neural connectivity in attention-deficit/hyperactivity disorder and alcohol use disorder studied using resting-state functional magnetic resonance imaging. *Alcohol Clin Exp Res.* (2021) 45:948–60. 10.1111/acer.14593 33690916

[39] Gerhardt S Luderer M Bumb J Sobanski E Moggi F Kiefer F Stop what you’re doing!-an fMRI study on comparisons of neural subprocesses of response inhibition in adhd and alcohol use disorder. *Front Psychiatry.* (2021) 12:691930. 10.3389/fpsyt.2021.691930 34603097 PMC8481878

[40] Brunkhorst-Kanaan N Libutzki B Reif A Larsson H McNeill R Kittel-Schneider S. ADHD and accidents over the life span–a systematic review. *Neurosci Biobehav Rev.* (2021) 125:582–91. 10.1016/j.neubiorev.2021.02.002 33582234

[41] Assayag N Berger I Parush S Mell H Bar-Shalita T. Attention-deficit/hyperactivity disorder symptoms, sensation-seeking, and sensory modulation dysfunction in substance use disorder: a cross sectional two-group comparative study. *Int J Environ Res Public Health.* (2022) 19:5. 10.3390/ijerph19052541 35270233 PMC8909105

[42] MacDonald B Sadek J. Naturalistic exploratory study of the associations of substance use on ADHD outcomes and function. *BMC Psychiatry.* (2021) 21:251. 10.1186/s12888-021-03263-6 33980212 PMC8117494

[43] Francx W Zwiers M Mennes M Oosterlaan J Heslenfeld D Hoekstra P White matter microstructure and developmental improvement of hyperactive/impulsive symptoms in attention-deficit/hyperactivity disorder. *J Child Psychol Psychiatry.* (2015) 56:1289–97. 10.1111/jcpp.12379 25581343 PMC4499023

[44] Posner J Polanczyk G Sonuga-Barke E. Attention-deficit hyperactivity disorder. *Lancet.* (2020) 395:450–62. 10.1016/S0140-6736(19)33004-1 31982036 PMC7880081

[45] Franke B Michelini G Asherson P Banaschewski T Bilbow A Buitelaar J Live fast, die young? A review on the developmental trajectories of ADHD across the lifespan. *Eur Neuropsychopharmacol J Eur Coll Neuropsychopharmacol.* (2018) 28:1059–88. 10.1016/j.euroneuro.2018.08.001 30195575 PMC6379245

[46] Zulauf C Sprich S Safren S Wilens T. The complicated relationship between attention deficit/hyperactivity disorder and substance use disorders. *Curr Psychiatry Rep.* (2014) 16:436. 10.1007/s11920-013-0436-6 PMC4414493 24526271

[47] Konstenius M Leifman A van Emmerik-van Oortmerssen K van de Glind G Franck J Moggi F Childhood trauma exposure in substance use disorder patients with and without ADHD. *Addict Behav.* (2017) 65:118–24. 10.1016/j.addbeh.2016.10.016 27816036 PMC5518307

[48] Maharjan S Amjad Z Abaza A Vasavada A Sadhu A Valencia C Executive dysfunction in patients with alcohol use disorder: a systematic review. *Cureus.* (2022) 14:e29207. 10.7759/cureus.29207 36258974 PMC9573267

[49] Stanford M Mathias C Dougherty D Lake S Anderson N Patton J. Fifty years of the barratt impulsiveness scale: an update and review. *Personality Indiv Diff.* (2009) 47:385–95. 10.1016/j.paid.2009.04.008

[50] Ivanov I Parvaz M Velthorst E Shaik R Sandin S Gan G Substance use initiation, particularly alcohol, in drug-naive adolescents: possible predictors and consequences from a large cohort naturalistic study. *J Am Acad Child Adoles Psychiatry.* (2021) 60:623–36. 10.1016/j.jaac.2020.08.443 33011213

[51] Elkins I Saunders G Malone S Keyes M McGue M Iacono W. Associations between childhood ADHD, gender, and adolescent alcohol and marijuana involvement: a causally informative design. *Drug Alcohol Dep.* (2018) 184:33–41. 10.1016/j.drugalcdep.2017.11.011 29402677 PMC5818293

[52] Haden S Shiva A. Trait impulsivity in a forensic inpatient sample: an evaluation of the barratt impulsiveness scale. *Behav Sci Law.* (2008) 26:675–90. 10.1002/bsl.820 19039789

[53] D’Alessandro A Bendimerad P. Executives functions in co-occuring adult attention deficit hyperactivity disorder and alcohol use disorder. *Eur Psychiatry.* (2021) 64:S243. 10.1192/j.eurpsy.2021.652

[54] Domínguez-Salas S Díaz-Batanero C Lozano-Rojas O Verdejo-García A. Impact of general cognition and executive function deficits on addiction treatment outcomes: systematic review and discussion of neurocognitive pathways. *Neurosci Biobehav Rev.* (2016) 71:772–801. 10.1016/j.neubiorev.2016.09.030 27793597

[55] van Emmerik-van Oortmerssen K Blankers M Vedel E Kramer F Goudriaan A van den Brink W Prediction of drop-out and outcome in integrated cognitive behavioral therapy for ADHD and SUD: results from a randomized clinical trial. *Addict Behav.* (2020) 103:106228. 10.1016/j.addbeh.2019.106228 31838443

[56] Zhang S Qiu S Pan M Zhao M Zhao R Liu L Adult ADHD, executive function, depressive/anxiety symptoms, and quality of life: a serial two-mediator model. *J Affect Disord.* (2021) 293:97–108. 10.1016/j.jad.2021.06.020 34175595

[57] Mowlem F Agnew-Blais J Pingault J Asherson P. Evaluating a scale of excessive mind wandering among males and females with and without attention-deficit/hyperactivity disorder from a population sample. *Sci Rep.* (2019) 9:3071. 10.1038/s41598-019-39227-w 30816143 PMC6395591

[58] Galéra C Orriols L M’Bailara K Laborey M Contrand B Ribéreau-Gayon R Mind wandering and driving: responsibility case-control study. *BMJ.* (2012) 345:e8105. 10.1136/bmj.e8105 23241270 PMC3521876

[59] Jana S Aron A. Mind wandering impedes response inhibition by affecting the triggering of the inhibitory process. *Psychol Sci.* (2022) 33:1068–85. 10.1177/09567976211055371 35699435 PMC9437729

[60] Mick E Faraone S Spencer T Zhang H Biederman J. Assessing the validity of the quality of life enjoyment and satisfaction questionnaire—short form in adults with ADHD. *J Attent Dis.* (2008) 11:504–9. 10.1177/1087054707308468 17934183

[61] Ugochukwu C Bagot K Delaloye S Pi S Vien L Garvey T The importance of quality of life in patients with alcohol abuse and dependence. *Harvard Rev Psychiatry.* (2013) 21:1–17. 10.1097/HRP.0b013e31827fd8aa 23656759

[62] Johnson B Ait-Daoud N Akhtar F Ma J. Oral topiramate reduces the consequences of drinking and improves thequality of life of alcohol-dependent individuals: a randomized controlled trial. *Arch General Psychiatry.* (2004) 61:905–12. 10.1001/archpsyc.61.9.905 15351769

[63] Coghill D Banaschewski T Soutullo C Cottingham M Zuddas A. Systematic review of quality of life and functional outcomes in randomized placebo-controlled studies of medications for attention-deficit/hyperactivity disorder. *Eur Child Adoles Psychiatry.* (2017) 26:1283–307. 10.1007/s00787-017-0986-y 28429134 PMC5656703

[64] Hagman B Falk D Litten R Koob G. Defining recovery from alcohol use disorder: development of an NIAAA research definition. *Am J Psychiatry.* (2022) 2022:aiaj21090963. 10.1176/appi.ajp.21090963 35410494

